# Adult mortality and probable cause of death in rural northern Malawi in the era of HIV treatment

**DOI:** 10.1111/j.1365-3156.2012.02929.x

**Published:** 2012-07-30

**Authors:** Menard Chihana, Sian Floyd, Anna Molesworth, Amelia C Crampin, Ndoliwe Kayuni, Alison Price, Basia Zaba, Andreas Jahn, Hazzie Mvula, Albert Dube, Bagrey Ngwira, Judith R Glynn, Neil French

**Affiliations:** 1 Karonga Prevention StudyChilumba, Malawi; 2 London School of Hygiene and Tropical MedicineLondon, UK

**Keywords:** HIV, cause of death, Africa, verbal autopsy

## Abstract

**Objectives:**

Developing countries are undergoing demographic transition with a shift from high mortality caused by communicable diseases (CD) to lower mortality rates caused by non-communicable diseases (NCD). HIV/AIDS has disrupted this trend in sub-Saharan Africa. However, in recent years, HIV-associated mortality has been reduced with the introduction of widely available antiretroviral therapy (ART). Side effects of ART may lead to increased risk of cardiovascular diseases, raising the prospects of an accelerated transition towards NCD as the primary cause of death. We report population-based data to investigate changes in cause of death owing to NCD during the first 4 years after introduction of HIV treatment.

**Methods:**

We analysed data from a demographic surveillance system in Karonga district, Malawi, from September 2004 to August 2009. ART was introduced in mid-2005. Clinician review of verbal autopsies conducted 2–6 weeks after a death was used to establish a single principal cause of death.

**Results:**

Over the entire period, there were 905 deaths, AIDS death rate fell from 505 to 160/100 000 person-years, and there was no evidence of an increase in NCD rates. The proportion of total deaths attributable to AIDS fell from 42% to 17% and from NCD increased from 37% to 49%.

**Discussion:**

Our findings show that 4 years after the introduction of ART into HIV care in Karonga district, all-cause mortality has fallen dramatically, with no evidence of an increase in deaths owing to NCD.

## Introduction

Many developing countries including countries in sub-Saharan Africa are undergoing demographic transition with a shift from high mortality caused by communicable diseases (CD) to lower mortality rates caused by non-communicable diseases (NCD). In 2005, it was estimated that about 35 million people died of NCD worldwide and around 80% of these occurred in low- and middle-income countries (WHO/World Economic Forum report 2008). It is also predicted that by 2020, NCD will cause 70% of all deaths in the developing world. In Africa, this transition has been dramatically affected by HIV infection. The rise in AIDS mortality and the re-emergence of other infectious diseases and associated increase in mortality heightened the concern that countries that are still in the process of transition might be faced with a double burden of diseases, infectious and non-infectious.

The contribution of HIV infection to mortality has been reduced in recent years with the introduction of widely available antiretroviral therapy (ART). In northern Malawi (Jahn *et al.* 2008; Floyd *et al.* 2010) and elsewhere (Herbst *et al.* 2009; Reniers *et al.* 2009; Mwagomba *et al.* 2010), the population-level impacts of this intervention have been shown to be large for both all-cause and AIDS mortality. With decreased AIDS mortality and the disease reduced to the level of a chronic illness by the availability of ART, a high proportion of people will now live longer. It is thus anticipated that there will be a shift in mortality patterns to more deaths owing to NCD (Mayosi *et al.* 2009). How quickly and how large will be the change to NCD mortality in African countries badly affected by HIV but with falling HIV-associated mortality is unclear. ART use is associated with a range of side effects particularly so with the older agents in widespread use in African treatment programmes. Among these side effects, derangement of lipid and glucose metabolism and impairment of renal function with consequent alterations in blood pressure may create a particular risk for cardiovascular disease (Brown *et al.* 2005; Mocroft *et al.* 2007; Makinson *et al.* 2008; Crane *et al.* 2011).

Malawi is one of the more severely HIV-affected African nations with an adult HIV prevalence of around 14%, but has also been one of the most successful in responding to the needs for treatment. In 2004, the country introduced a nationwide free ART treatment programme and the country is already benefiting from the fruits of a public health approach to ART delivery. We have been following a rural population in Karonga district in northern Malawi over the past 8 years and have reported the changes in overall and HIV-associated mortality (Jahn *et al.* 2008; Floyd *et al.* 2010). By August 2009, 4 years into the ART programme in Karonga district, approximately 63% of the need for ART was estimated to have been met in the local population (Floyd *et al.* 2010). We now describe details of the non-communicable causes of death to investigate whether there is evidence of an increasing burden of NCD during this initial period of ART introduction.

## Methods

### Study area

The Karonga Demographic Surveillance site (DSS) was set up in August 2002 with a baseline census to register all individuals and households in the study area (Jahn *et al.* 2007). The baseline census ended in August 2004, at that time a continuous registration system (CRS) for demographic events has been operational in the whole of the DSS area. Located in the southern part of Karonga district, the DSS is between latitudes 10.38° S and 10.50° S and longitudes 34.08° E and 34.27° E and is bordered by Lake Malawi in the east, Nyika National Park in the south and west, and the north demarcation follows village boundaries. The DSS has a population of about 33 500 individuals based on 2008 mid-year population estimates, most of whom are rural dwellers that depend on subsistence farming and fish from the lake as a source of their livelihood. The area has two ART clinics: Buyu, which is 70 km from the DSS, became operational in July 2005 and Mwabi, which is located within the DSS area, became operational in October 2006.

### Data sources

A system of village key informants is used to record and report births and deaths taking place in the area each month, and an annual census updates household composition and migration as well as whole household change, e.g. formation of a new household, in-migration of a whole household from outside the DSS and movement of a whole household within the DSS. Each key informant is responsible for recording and reporting events for a single geographically defined reporting cluster, which on average consists of 35 households. After a key informant report, an interviewer will visit the household to record a birth or a death (Jahn *et al.* 2007). Since September 2007, HIV testing has been offered door-to-door to all individuals aged 15 years and older in the DSS.

When a death is reported, after allowing at least 2 weeks of mourning, a medically trained interviewer visits the deceased’s household to fill a semi-structured verbal autopsy (VA) form using a method described previously (INDEPTH Network 2003). Verbal consent is sought before the interview. Information is gathered from a person who was present during the deceased’s final illness or a close relative. The completed VA forms are given to two clinicians to review independently, each assigning a cause of death from a pre-defined hierarchical list. For example, if a specific condition cannot be assigned but is believed to be communicable disease, then CD-unspecified can be assigned. The HIV status of the deceased person, if known from previous quality-controlled and verified karonga prevention study (KPS) testing, is made available to reviewers but HIV-infected individuals are not automatically assumed to have died of HIV-related illnesses. If there is a discrepancy in the diagnosis given by the two independent reviewers, a third review is performed by another clinician who reviews the verbal autopsy alongside the coding and comments of the first reviews to provide a final decision. When there is no significant pathology to explain the cause of death, such that not even a main grouping can be assigned, the cause of death is classified as unknown. Deaths where no information from the VA form was available or specific information was missing on the form were classified as missing data.

### Analysis

The analysis presented here covers the period from 1 September 2004, the date by which active vital events reporting had been launched in the whole DSS area to 31 August 2009. Person-years of observation (PYO) were calculated from the 1 September 2004, or the date of migration into the DSS area if this came later, until the earliest of date of death, out-migration from the DSS, or the end 31 of August 2009. The person-years (time at risk) were used as a denominator in the calculation of all mortality rates and expressed as deaths per 100 000 PYO. Analysis was performed using stata version 9 (StataCorp, College Station, TX, USA), and graphs were performed in Microsoft Excel 2007. For analysis of all-cause mortality, all deaths in the population are included in the numerator for the calculation of mortality rates and individuals were censored at the time of their death. For analysis of *cause-specific* mortality, individuals who died because of a different or unknown cause, or with missing data on the verbal autopsy form, were not included in the numerator for the calculation of mortality rates, but instead were censored from the analysis on the date they died.

Year-on-year comparison of rates, following the DSS survey round, which starts in September and ends in August, was used to measure trends in mortality, with September 2004 to August 2005 as the base year. Poisson regression was used to calculate rate ratios (RRs) for the effect of time period, overall and stratified on age group and sex. All-cause and category-specific mortality rates were compared to measure the impact of ART provision on mortality, with tests for trend using likelihood ratio tests. The causes of death were grouped as CD, further divided into AIDS and other CD, NCD, external causes, unknown causes and missing data. Three age groups were considered in the analysis – young adults 15–44 years, middle-aged adults 45–59 years and older adults 60 or more years.

Ethical approval for the study was obtained from the National Health Sciences Research Committee of Malawi and the Ethics Committee of the London School of Hygiene and Tropical Medicine.

## Results

The CRS area has an adult population of 17 373 individuals based on 2008 mid-year population estimates with 8124 (46.8%) males and 9249 (53.2%) females. Individuals aged 15–44 years numbered 13 283, 45–59 years 2180 and 60 or more years 1910. From September 2004 to August 2009, a total of 905 adult deaths were reported in 81 870 PYO giving a crude death rate of 1105/100 000 PYO (Table 1). For 75 deaths (8.3%), the cause was either classified as unknown or missing data (Table 2). Of these, 16 were among 15- to 44-year olds, eight among 45- to 59-year olds and 51 among individuals aged 60 or more years. Of all the people who died, 428 (47.3%) were males. 370 (40.9%) of the total deaths were among 15- to 44-year olds in 62 810 PYO giving a death rate of 589/100 000 PYO (95% CI 532–652); 165 (18.2%) deaths were among 45- to 59-year olds, 10 140 PYO with a death rate of 1627/100 000 PYO (95% CI 1397–1895); and 370 (40.9%) deaths, were among individuals aged 60 or more years old, from 8930 PYO, with a death rate 4146/100 000 PYO (95% CI 3744–4590). In summary, all-cause mortality rate increased sharply with age group, for 92% of deaths a cause was assigned, and most of the deaths of unknown or un-specifiable cause were among adults aged 60 or more years old.

**Table 1:** Probable cause of death of the 905 total adult deaths recorded in the Karonga Demographic Surveillance site during the period 1 September 2004–31 August 2009

	September 4–August 5	September 5–August 6	September 6–August 7	September 7–August 8	September 8–August 9	
						
	Year 1	Year 2	Year 3	Year 4	Year 5	Total
						
Probable cause of death	*n* (%)	*n* (%)	*n* (%)	*n* (%)	*n* (%)	*n* (%)
Communicable disease						
HIV/AIDS	81 (42.0)	76 (35.7)	49 (29.3)	45 (23.6)	24 (16.9)	275 (30.0)
TB or HIV/AIDS	1 (0.5)	2 (0.9)	0 (0)	2 (1.1)	1 (0.7)	6 (0.7)
TB	4 (2.1)	6 (2.8)	2 (1.2)	7 (3.7)	3 (2.1)	22 (2.4)
Pneumonia	3 (1.6)	7 (3.3)	4 (2.4)	7 (3.7)	6 (4.3)	27 (3)
Hepatitis	2 (1.0)	2 (0.9)	0 (0)	0 (0)	2 (1.4)	6 (0.7)
Malaria	1 (0.5)	0 (0)	3 (1.8)	0 (0)	2 (1.4)	6 (0.7)
Meningitis	3 (1.6)	6 (2.8)	7 (4.2)	3 (1.6)	3 (2.1)	22 (2.4)
Diarrhoeal	2 (1.0)	4 (1.9)	6 (3.6)	4 (2.1)	3 (2.1)	19 (2.1)
Acute febrile disease other	3 (1.6)	5 (2.4)	11 (6.6)	4 (2.1)	8 (5.7)	31 (3.4)
Other	2 (1)	1 (0.5)	1 (0.6)	1 (0.5)	0 (0)	5 (0.6)
Total	102 (52.9)	109 (51.2)	83 (49.7)	73 (38.4)	52 (36.7)	419 (46.0)
Non-communicable diseases (NCD)						
CV unspecifiable/other	1 (0.5)	0 (0)	0 (0)	3 (1.6)	1 (0.7)	5 (0.6)
Hypertension	7 (3.7)	9 (4.2)	2 (1.2)	2 (1.1)	1 (0.7)	21 (2.3)
Congestive cardiac failure	11 (5.7)	13 (6.1)	6 (3.6)	7 (3.7)	7 (4.9)	44 (4.9)
Ischaemic heart disease	1 (0.5)	0 (0)	0 (0)	1 (0.5)	0 (0)	2 (0.2)
Cerebrovascular disease	8 (4.2)	9 (4.2)	13 (7.8)	16 (8.4)	9 (6.3)	55 (6.1)
Gastrointestinal	12 (6.3)	9 (4.2)	8 (4.8)	15 (7.9)	17 (12)	61 (6.7)
Maternal	4 (2.1)	5 (2.4)	5 (3)	8 (4.2)	3 (2.1)	25 (2.8)
Cancer	10 (5.2)	8 (3.8)	8 (4.8)	13 (6.8)	9 (6.3)	48 (5.3)
Diabetes	3 (1.6)	6 (2.8)	5 (3)	3 (1.6)	9 (6.3)	26 (2.9)
Respiratory	1 (0.5)	3 (1.4)	2 (1.2)	3 (1.6)	4 (2.8)	13 (1.4)
Central nervous system	3 (1.6)	2 (0.9)	7 (4.2)	2 (1.1)	2 (1.4)	16 (1.8)
Genito-urinary	5 (2.6)	2 (0.9)	0 (0)	5 (2.6)	3 (2.1)	15 (1.7)
Anaemia	2 (1)	2 (0.9)	1 (0.6)	4 (2.1)	1 (0.7)	10 (1.1)
Nutritional	2 (1)	0 (0)	3 (1.8)	0 (0)	1 (0.7)	6 (0.7)
Other NCD	1 (0.5)	5 (2.4)	2 (1.2)	7 (3.7)	2 (1.4)	17 (1.9)
Total	71 (37)	73 (34.2)	62 (37.2)	89 (46.9)	69 (48.4)	364 (40.4)
External causes						
Accidental	5 (2.6)	6 (2.8)	5 (3)	8 (4.2)	4 (2.8)	28 (3.1)
Suicide	2 (1)	0 (0)	2 (1.2)	2 (1.1)	1 (0.7)	7 (0.8)
Assault	0 (0)	1 (0.5)	0 (0)	2 (1.1)	1 (0.7)	4 (0.4)
Unknown intent/other	1 (0.5)	2 (0.9)	1 (0.6)	1 (0.5)	3 (2.1)	8 (0.9)
Total	8 (4.1)	9 (4.2)	8 (4.8)	13 (6.9)	9 (6.3)	47 (5.2)
Cause of death unknown/missing data						
Unknown	7 (3.7)	17 (8)	12 (7.2)	7 (3.7)	4 (2.8)	47 (5.2)
Missing data	4 (2.1)	5 (2.4)	2 (1.2)	9 (4.7)	8 (5.7)	28 (3.1)
Total	11 (5.8)	22 (10.4)	14 (8.4)	16 (8.4)	12 (8.5)	75 (8.3)
Overall total	192 (100)	213 (100)	167 (100)	191 (100)	142 (100)	905 (100)

**Table 2:** Mortality rates per 100 000 person-years for principal causes of death by reporting year, overall and broken by gender and age group

		All cause		AIDS		Other communicable disease		Non-communicable disease	
					
	Person-years of observation	*n*	Rate (95% CI)	*n*	Rate (95% CI)	*n*	Rate (95% CI)	*n*	Rate (95% CI)
Overall									
Year 1	16 050	192	1197 (1039–1378)	81	505 (406–627)	21	131 (85–201)	71	443 (351–558)
Year 2	16 650	213	1280 (1119–1464)	76	457 (365–572)	33	198 (141–279)	73	439 (349–551)
Year 3	17 020	167	981 (843–1142)	49	288 (218–381)	34	199 (143–280)	62	364 (284–467)
Year 4	17 120	191	1115 (968–1285)	45	263 (196–352)	28	163 (113–237)	89	520 (422–640)
Year 5	15 040	142	944 (801–1113)	24	160 (107–238)	28	186 (129–270)	69	459 (362–581)
Total	81 870	905	1105 (1036–1180)	275	336 (298–378)	144	176 (149–207)	364	445 (401–493)
By sex									
Men									
Year 1	7540	90	1194 (971–1469)	33	438 (311–616)	11	146 (81–264)	33	438 (311–616)
Year 2	7800	100	1281 (1053–1559)	35	449 (322–625)	18	231 (145–366)	30	384 (269–550)
Year 3	7970	73	916 (728–1151)	26	326 (222–479)	15	188 (113–312)	18	226 (142–358)
Year 4	8020	90	1122 (912–1379)	25	312 (211–461)	13	162 (94–279)	36	449 (324–622)
Year 5	7020	75	1068 (852–1340)	13	185 (108–319)	16	228 (140–372)	34	484 (346–678)
Total	38 360	428	1116 (1015–1227)	132	334 (290–408)	73	190 (151–239)	151	394 (336–462)
Women									
Year 1	8510	102	1199 (987–1455)	48	564 (425–748)	10	118 (63–218)	38	447 (325–614)
Year 2	8840	113	1278 (1063–1537)	41	464 (341–630)	15	170 (102–281)	43	486 (261–656)
Year 3	9050	94	1039 (849–1272)	23	254 (169–383)	19	210 (134–329)	44	486 (362–654)
Year 4	9100	101	1110 (913–1349)	20	220 (142–341)	15	165 (99–273)	53	582 (445–762)
Year 5	8020	67	835 (657–1061)	11	137 (76–248)	12	150 (85–263)	35	436 (313–608)
Total	43 520	477	1096 (1002–1199)	143	329 (279–387)	71	163 (129–206)	213	490 (428–560)
By age									
15–44									
Year 1	12 280	82	668 (538–829)	53	432 (330–565)	8	65 (33–130)	17	139 (86–223)
Year 2	12 800	93	727 (593–890)	51	398 (303–524)	15	117 (71–194)	16	125 (77–204)
Year 3	13 120	71	541 (429–683)	32	244 (173–345)	15	114 (69–190)	19	145 (92–227)
Year 4	13 120	72	549 (436–692)	30	229 (160–327)	11	84 (46–151)	21	160 (104–246)
Year 5	11 500	52	452 (345–593)	16	139 (85–227)	10	87 (47–162)	17	148 (91–238)
Total	62 810	370	589 (532–652)	182	290 (251–335)	59	94 (73–121)	90	143 (117–176)
45–59									
Year 1	2050	33	1610 (1144–2264)	20	975 (629–1500)	2	98 (24–390)	7	341 (163–716)
Year 2	2020	43	2129 (1579–2870)	21	1000 (678–1600)	8	396 (198–792)	12	594 (337–1046)
Year 3	2050	30	1466 (1025–2096)	13	635 (369–1100)	4	195 (73–521)	8	391 (195–781)
Year 4	2130	35	1646 (1182–2292)	13	611 (355–1100)	5	235 (98–565)	14	658 (390–1111)
Year 5	1900	24	1264 (847–1886)	7	369 (176–773)	5	263 (110–633)	8	421 (211–843)
Total	10 140	165	1627 (1397–1895)	74	730 (581–916)	24	237 (159–353)	49	483 (365–639)
60+									
Year 1	1720	77	4485 (3588–5608)	8	466 (223–932)	11	641 (355–1157)	47	2738 (2057–3644)
Year 2	1830	77	4214 (3370–5269)	4	219 (82–583)	10	547 (294–1017)	45	2463 (1839–3298)
Year 3	1850	66	3558 (2795–4529)	4	216 (81–575)	15	809 (488–1341)	35	1887 (1355–2628)
Year 4	1880	84	4464 (847–1886)	2	106 (27–425)	12	638 (362–1123)	54	2869 (2198–3746)
Year 5	1640	66	4013 (3153–5108)	1	61 (9–431)	13	791 (459–1361)	44	2676 (1991–3595)
Total	8930	370	4146 (3744–4590)	19	213 (136–334)	61	683 (532–878)	225	2521 (2212–2873)

Overall, CD were the largest cause of death: 419 (46%) of the total deaths 205 (48.9%) of which were males. AIDS was the single leading cause of death contributing 275 (65.6%) deaths to this category and (30%) overall. Pneumonia was the second most important cause of communicable disease deaths followed by TB (Table 1). Among non-communicable causes of death, the most common cause was classified as gastrointestinal diseases (incorporating peptic ulcer, liver cirrhosis and acute abdomen including obstruction within this classification), 6.7% (*n* = 61). Of the classifiable clinical syndromes, cerebrovascular disease, congestive cardiac failure, diabetes and hypertension were the major classified types (Table 1). Deaths owing to external causes only contributed a small proportion to the overall adult mortality: 47 deaths (5.2%) of which 38 (80.9% of all deaths from external clauses) were males – 28 (3.1%) were as a result of accidents whilst only 7 (0.8%) were identified as owing to suicide.

The proportion of deaths attributed to other communicable and non-communicable causes and AIDS varied by age. Among 15- to 44-year olds, 16% of deaths were attributed to other communicable causes, 24.3% to a non-communicable cause and 49.2% to AIDS. The corresponding figures among 45- to 59-year olds were 14.6%, 29.7% and 44.9%, and among individuals aged 60 years or more were 16.5%, 60.8% and 5.1% (Table 4).

**Table 4:** Number (and percentage) of deaths attributable to each broad cause of death category among adults for each survey year overall and broken down by gender and age group

	AIDS	Other communicable	Non-communicable	External	Missing/unspecified	Total
Overall						
Year 1	81 (42.2)	21 (10.9)	71 (37.0)	8 (4.2)	11 (5.7)	192
Year 2	76 (35.7)	33 (15.5)	73 (34.3)	8 (4.2)	22 (10.3)	213
Year 3	49 (29.3)	34 (20.4)	62 (37.1)	8 (4.8)	14 (8.4)	167
Year 4	45 (23.6)	28 (14.7)	89 (46.6)	13 (6.8)	16 (8.4)	191
Year 5	24 (16.9)	28 (19.7)	69 (48.6)	9 (6.3)	12 (8.5)	142
Total	275 (30.4)	144 (15.9)	364 (40.2)	47 (5.2)	75 (8.3)	905
By sex						
Men						
Year 1	33 (36.7)	11 (12.2)	33 (36.7)	7 (7.8)	6 (6.7)	90
Year 2	35 (35.0)	18 (18.0)	30 (30.0)	7 (7.0)	10 (10.0)	100
Year 3	26 (35.6)	15 (20.6)	18 (24.6)	7 (9.6)	7 (9.6)	73
Year 4	25 (27.8)	13 (14.4)	36 (40.0)	11 (12.2)	5 (5.6)	90
Year 5	13 (17.3)	16 (21.3)	34 (45.3)	6 (8.0)	6 (8.0)	75
Total	132 (30.8)	73 (17.1)	151 (35.3)	38 (8.9)	34 (7.9)	428
Women						
Year 1	48 (47.1)	10 (9.8)	38 (37.3)	1 (1.0)	5 (4.9)	102
Year 2	41 (36.3)	15 (13.3)	43 (38.1)	2 (1.8)	12 (10.6)	113
Year 3	23 (24.5)	19 (20.2)	44 (46.8)	1 (1.1)	7 (7.5)	94
Year 4	20 (19.8)	15 (14.9)	53 (52.5)	2 (2.0)	11 (10.9)	101
Year 5	11 (16.2)	12 (17.9)	35 (52.2)	3 (4.5)	6 (9.0)	67
Total	143 (30.0)	71 (14.9)	213 (44.7)	9 (1.9)	41 (8.6)	477
By age						
15–44						
Year 1	53 (64.6)	8 (9.8)	17 (20.7)	3 (3.7)	1 (1.2)	82
Year 2	51 (54.8)	15 (16.1)	16 (17.2)	6 (6.5)	5 (5.4)	93
Year 3	32 (45.1)	15 (21.1)	19 (26.8)	3 (4.2)	2 (2.8)	71
Year 4	30 (41.7)	11 (15.3)	21 (29.2)	5 (7.0)	5 (6.9)	72
Year 5	16 (30.8)	10 (19.2)	17 (32.7)	6 (11.5)	3 (5.8)	52
Total	182 (49.2)	59 (16.0)	90 (24.3)	23 (6.2)	16 (4.3)	370
45–59						
Year 1	20 (60.6)	2 (6.1)	7 (21.2)	3 (9.1)	1 (3.1)	33
Year 2	21 (48.8)	8 (18.6)	12 (27.9)	1 (2.3)	1 (2.3)	43
Year 3	13 (43.3)	4 (13.3)	8 (26.7)	2 (6.7)	3 (10.0)	30
Year 4	13 (37.1)	5 (14.3)	14 (40.0)	3 (8.6)	0 (0.0)	35
Year 5	7 (29.2)	5 (20.8)	8 (33.3)	1 (4.2)	3 (12.5)	24
Total	74 (44.9)	24 (14.6)	49 (29.7)	10 (6.1)	8 (4.9)	165
60+						
Year 1	8 (10.4)	11 (14.3)	47 (61.0)	2 (2.6)	9 (11.7)	77
Year 2	4 (5.2)	10 (13.0)	45 (58.4)	2 (2.6)	16 (20.8)	77
Year 3	4 (6.1)	15 (22.7)	35 (53.0)	3 (4.6)	9 (13.6)	66
Year 4	2 (2.4)	12 (14.3)	54 (64.3)	5 (6.0)	11 (13.1)	84
Year 5	1 (1.5)	13 (19.7)	44 (66.7)	2 (3.0)	6 (9.1)	66
Total	19 (5.1)	61 (16.5)	225 (60.8)	14 (3.8)	51 (13.8)	370

### Mortality trends

The total mortality rate fell from 1197/100 000 PYO in 2004–05 to 944/100 000 PYO in 2008–09, a decline of about 21% (95% CI 2–36%), *P* = 0.03 (Tables 2 and 3). This fall was seen in both men and women, and the fall was larger among individuals aged 15–59 years than among individuals aged 60 or more years.

**Table 3:** Mortality rate ratios (RR) for different causes of death, relative to the rate in Year 1 (2004–5). Broken down by gender and age group

	All cause	AIDS	Other communicable disease	Non-communicable disease
				
	RR (95% CI)	RR (95% CI)	RR (95% CI)	RR (95% CI)
Overall				
Year 1	Reference			
Year 2	1.07 (0.88–1.30)	0.90 (0.66–1.24)	1.52 (0.88–2.62)	0.99 (0.72–1.37)
Year 3	0.82 (0.67–1.01)	0.57 (0.40–0.81)	1.53 (0.89–2.63)	0.82 (0.59–1.16)
Year 4	0.93 (0.76–1.14)	0.52 (0.36–0.75)	1.25 (0.71–2.20)	1.18 (0.86–1.60)
Year 5	0.79 (0.64–0.98)	0.32 (0.20–0.50)	1.42 (0.81–2.51)	1.04 (0.74–1.44)
By sex				
Men				
Year 1	Reference			
Year 2	1.07 (0.81–1.43)	1.02 (0.64–1.65)	1.58 (0.75–3.35)	0.88 (0.54–1.44)
Year 3	0.77 (0.56–1.04)	0.74 (0.45–1.25)	1.29 (0.59–2.81)	0.52 (0.29–0.92)
Year 4	0.94 (0.70–1.26)	0.71 (0.42–1.20)	1.11 (0.50–2.48)	1.02 (0.64–1.64)
Year 5	0.89 (0.66–1.22)	0.42 (0.22–0.80)	1.56 (0.72–3.36)	1.11 (0.68–1.79)
Women				
Year 1	Reference			
Year 2	1.07 (0.82–1.39)	0.82 (0.54–1.25)	1.44 (0.65–3.21)	1.09 (0.70–1.69)
Year 3	0.87 (0.66–1.15)	0.45 (0.40–0.81)	1.19 (0.83–3.84)	1.09 (0.71–1.68)
Year 4	0.93 (0.70–1.26)	0.39 (0.23–0.66)	1.40 (0.63–3.12)	1.30 (0.86–1.98)
Year 5	0.70 (0.51–0.95)	0.24 (0.13–0.47)	1.27 (0.55–2.95)	0.98 (0.62–1.55)
By age				
15–44				
Year 1	Reference			
Year 2	1.09 (0.81–1.46)	0.92 (0.63–1.36)	1.80 (0.76–4.24)	0.90 (0.46–1.79)
Year 3	0.81 (0.59–1.11)	0.57 (0.36–0.88)	1.76 (0.74–4.14)	1.05 (0.54–2.01)
Year 4	0.82 (0.60–1.13)	0.53 (0.34–0.83)	1.29 (0.52–3.20)	1.16 (0.61–2.19)
Year 5	0.68 (0.48–0.96)	0.32 (0.18–0.56)	1.33 (0.53–3.38)	1.07 (0.55–2.09)
45–59				
Year 1	Reference			
Year 2	1.32 (0.84–2.08)	0.65 (0.32–1.31)	4.06 (0.86–19.12)	1.74 (0.69–4.42)
Year 3	0.91 (0.56–1.49)	0.65 (0.32–1.31)	2.00 (0.37–10.94)	1.14 (0.42–3.16)
Year 4	1.02 (0.64–1.65)	0.63 (0.31–1.26)	2.41 (0.47–12.40)	1.93 (0.78–4.78)
Year 5	0.79 (0.46–1.33)	0.38 (0.16–0.89)	2.71 (0.52–13.92)	1.23 (0.45–3.40)
60+				
Year 1	Reference			
Year 2	0.94 (0.69–1.29)	0.47 (0.14–1.56)	0.85 (0.36–2.01)	0.90 (0.60–1.35)
Year 3	0.79 (0.57–1.10)	0.46 (0.14–1.54)	1.26 (0.58–2.75)	0.69 (0.44–1.07)
Year 4	1.00 (0.73–1.36)	0.23 (0.05–1.07)	1.00 (0.44–2.26)	1.05 (0.71–1.55)
Year 5	0.90 (0.64–1.24)	0.13 (0.02–1.04)	1.23 (0.55–2.75)	0.98 (0.65–1.47)

**Figure 1:**
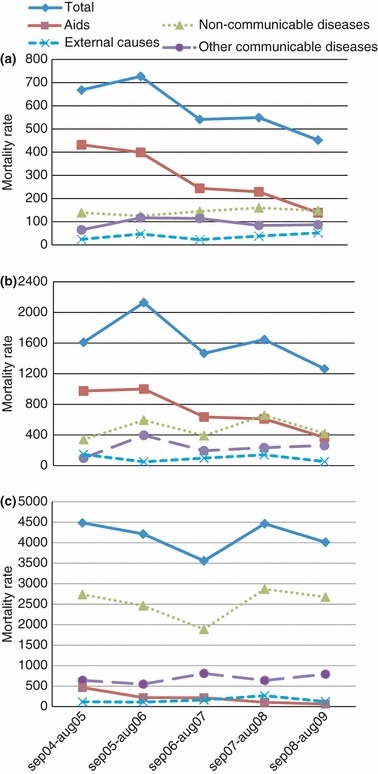
Trends in cause-specific adult mortality per 100 000 person-years, over 5 years from 2004 to 2009, broken down by age groups (a) Age 15–44, (b) 45–59, (c) 60+ years.

The AIDS mortality rate fell from 505 to 160/100 000 PYO in 2008–09, representing a 68% decrease and a rate ratio of 0.32 (95% CI 0.20–0.50), *P* < 0.0001 (Tables 2 and 3). The large falls in the AIDS mortality rate were seen in all age groups and in both men and women. Mortality attributable to the other communicable causes of death was higher after the baseline year, but did not rise further over time, and there were no consistent trends in NCD mortality rates (Table 3). The cause-specific trends in mortality, by age group, are summarised in Figure 1. In each age group, the only consistent trend over time was the decrease in AIDS mortality, with some fluctuations in the deaths attributable to NCD.

The substantial fall in AIDS mortality, with no corresponding rise in the mortality rate of other CD or NCD, resulted in a change in the proportion of total deaths that were owing to each broad cause of death. In both men and women, and all age groups, there was a fall in the proportion of total deaths that were attributed to AIDS – for example from 64.6% to 30.8% among individuals aged 15–44 years and from 60.6% to 29.2% among individuals aged 45–59 years. The proportion of total deaths attributed to other CD increased in both men and women, and in all age groups and overall from 10.9% to 19.7%. The proportion of total deaths attributed to NCD increased most among adults aged 15–59 years (Table 4).

## Discussion

During the period 2004–2009, which spanned a time of major change in the care of HIV-infected adults in Malawi, the all-cause mortality rate fell substantially among adults aged 15–59 years. There was a dramatic fall in AIDS mortality rate, with no rise in the mortality rate owing to NCD. As expected, the major benefits in reducing AIDS-related mortality were found in the younger age groups.

Fluctuations in cause of death may be owing to variations in verbal autopsy recording and coding (Quigley *et al.* 1999, 2000; Chandramohan *et al.* 2001; Quigley 2005; Lopman *et al.* 2006, 2010), a recognised problem in this approach. Changes in personell recording, the verbal autopsy data and clinicians undertaking coding have occurred during the period of this report but are unlikely to have led to major changes in classification. However, of particular note is that the proportion of the population whose HIV status was known increased over the course of the study as a consequence of house to house HIV testing undertaken by KPS – by 2009 approximately 80% of adults within the DSS had undergone an HIV test. These results were made available to the coding clinicians, which is likely to have influenced coding decisions. For instance, a less clear diagnosis may be more likely to be classified as AIDS when an HIV-positive test result is available, or excluded as AIDS when a recent HIV-negative test is available. Although this might have affected classification of cases of AIDS over time, it seems unlikely that this will have affected classification of NCD deaths.

We have measured no change in mortality rates attributable to NCD. A rise in NCD deaths would have been missed if NCD deaths were over classified in the earlier period and/or under classified in the later period. It is likely that such misclassification would be specific to certain clinical syndromes. For instance, an individual who dies from a cerebrovascular accident may be difficult to differentiate from an intra-cerebral AIDS-defining infection. Deaths as a consequence of the long-term consequences of hypertension or diabetes are less likely to be confused with HIV. The fact that no dramatic changes occurred by individual NCD clinical syndrome over time suggests that misclassification as a consequence of increased HIV testing has not been a major problem and supports the need to follow individual clinical syndrome level data.

A limitation of our current approach is the coding of one primary cause of death and not coding contributory conditions. This is likely to compromise our ability to identify NCD deaths in persons who are HIV infected and thus lead to under recognition of the complications of ART treatment. The VA tool was developed with an emphasis on estimating the burden of deaths attributable to illnesses for which public health interventions were available a decade ago (Chandramohan *et al.* 1998). The VA has a higher sensitivity for communicable disease conditions than non-communicable. With the evolution in HIV care and the conversion of this condition from a fatal to a chronic condition, it will become increasingly important to be able to identify the specific cause of death as it can no longer be expected that the primary cause of death will be an opportunistic infection and a direct consequence of HIV. We have now moved to restructure our verbal autopsy reporting to indicate primary and contributory causes of death, a change that will also allow a better understanding of the interaction between communicable disease and NCD relevant to HIV but also to conditions such as diabetes.

As we have shown previously, the group aged 60 or more years was the age group least affected by the AIDS pandemic (Floyd *et al.* 2010) and therefore had the least potential to benefit from the ART treatment programme. There was little overall change in mortality in this age group, although most of the deaths classified as unknown or missing data were in this age group. Although the high rates of NCD among adults aged 60 or more years are as expected, it is also notable that this age group has the highest rates of other CD, in particular owing to pneumonia, diarrhoeal disease, other acute febrile illness and tuberculosis. This is not surprising as old age is a predisposing factor for these conditions in all regions of the world. However, as the overall burden of death attributable to HIV declines, control of communicable disease in the older population represents a further target for attention in this region, with effective preventive strategies to control tuberculosis, pneumonia and influenza in particular, available for this age group.

The principal causes of death in the NCD group are consistent with findings from other developing countries, although comparative data from countries similar to Malawi in the south and eastern sub-Saharan African region are scarce (Mathers *et al.* 2009). Diseases related to the circulatory system, likely to be exacerbated by or directly related to hypertension, are the most important group. Measuring the extent of this problem and establishing measures to effectively control hypertension would seem to be a pressing health issue for this part of the world. Similarly, diabetes is a consistently reported problem and may increase as a side effect of ART. Effective methods for managing diabetes need to be developed. As illnesses that need chronic care, it may be possible to develop management strategies learning from the processes developed for the assessment of and delivery of care for HIV. Notable is the lack of deaths directly attributable to alcohol and tobacco abuse, areas identified for immediate action in the control of NCD (Beaglehole *et al.* 2011). In the case of alcohol, deaths attributable to its abuse may be difficult to identify in our population, but a proportion of the deaths attributed to external causes is highly likely to be alcohol related as well as cases of chronic liver disease. Tobacco use is low in this population (data not shown) and its contribution to cause of death is likely to be small at the present time.

Free ART treatment became available to the people of Karonga district in July 2005. At the end of this study period, we estimated 60% of the ART treatment need, based on the treatment criteria in effect in Malawi at that time, was being met in Karonga district. There is no evidence of an absolute increase in NCD mortality rates during this initial period of ART delivery, although the relative importance of these conditions has increased and will necessitate action to consider how these can best be prevented and managed in this setting. Over the longer term, this may change, with an increasing proportion of HIV-positive individuals on ART, on ART for a long duration, on treatment earlier as a consequence of changes to eligibility for ART along with broad underlying socio-demographic changes. It is also possible that the AIDS mortality rate could increase again, if retention in treatment programmes and treatment adherence are not maintained at a high level and/or drug resistance increases. Thus, continued monitoring of cause of death is essential and this will be best achieved through demographic surveys whilst national civil registration remains problematic. In addition, verbal autopsy coding will need to adapt to ensure that primary and contributory causes of death are identified to ensure multiple pathologies can be captured in an ageing HIV-infected population.
